# Patient uptake and outcomes following pharmacist-initiated referrals to general practitioners for asthma review

**DOI:** 10.1038/s41533-022-00315-6

**Published:** 2022-11-17

**Authors:** Sarah Serhal, Ines Krass, Lynne Emmerton, Bonnie Bereznicki, Luke Bereznicki, Sinthia Bosnic-Anticevich, Bandana Saini, Laurent Billot, Carol Armour

**Affiliations:** 1 Woolcock Institute of Medical Research, 431 Glebe Point Road, Glebe, NSW 2031 Australia; 2 The University of Sydney—Sydney Pharmacy School, A15, Science Rd, Camperdown, NSW 2006 Australia; 3 Curtin University—Curtin Medical School, Building 306, Brand Drive, Curtin University Bentley Campus, Perth, WA 6845 Australia; 4 University of Tasmania—Tasmanian School of Medicine, University of Tasmania, Private Bag 34, Hobart, TAS 7001 Australia; 5 University of Tasmania—School of Pharmacy and Pharmacology, University of Tasmania, Private Bag 26, Hobart, TAS 7001 Australia; 6 Central Sydney Area Health Service—Sydney, Camperdown, NSW Australia; 7 The George Institute, Level 5/1 King St, Newtown, NSW 2042 Australia; 8 University of New South Wales—Faculty of Medicine-Wallace Wurth Building-UNSW Sydney, 18 High St, Kensington, NSW 2052 Australia

**Keywords:** Health services, Health occupations, Preventive medicine

## Abstract

Uptake and outcomes of pharmacist-initiated general practitioner (GP) referrals for patients with poorly controlled asthma were investigated. Pharmacists referred at-risk patients for GP assessment. Patients were categorized as *action takers (*consulted their GP on pharmacist’s advice) or *action avoiders (*did not action the referral). Patient clinical data were compared to explore predictors of uptake and association with health outcomes. In total, 58% of patients (*n* = 148) received a GP referral, of whom 78% (*n* = 115) were *action takers*, and 44% (*n* = 50) reported changes to their asthma therapy. Patient rurality and more frequent pre-trial GP visits were associated with *action takers*. *Action takers* were more likely to have an asthma action plan (*P* = 0.001) at month 12, and had significantly more GP visits during the trial period (*P* = 0.034). Patient uptake of pharmacist-initiated GP referrals was high and led to GP review and therapy changes in patients with poorly controlled asthma.

## Introduction

A characteristic of an effective and efficient healthcare system is a synergistic relationship between its stakeholders^1^. Stakeholders must recognize and utilize each other’s unique skillsets and knowledge to increase accessibility to care, and strengthen the lines of defense against poor health within the population they serve^2^. In Australia, asthma management is primarily overseen by a general practitioner (GP); however, pharmacies are the most frequented healthcare venue for patients with asthma^3,4^.

A critical part of a pharmacist’s education, training, and practice focuses on their ability to recognize risk factors and warning signs that may indicate their patients require care and further assessment by a GP^5^. When a patient visits the pharmacy to collect asthma controller or reliever medicines, pharmacists have the opportunity, and competency to identify signs of worsening asthma and assist in triaging patients appropriately^2,6,7^. Referrals may be given verbally to the patient, in writing, or directly to the GP via immediate contact, dependent on the relationships between the pharmacist, the patient and their GP.

Community pharmacists’ surveillance also offers a safety net within primary care to overcome asthma patients’ underestimation of the severity of their condition, and encourage patients to seek help^8^. Underestimation of asthma severity can lead to complacency, delays in seeking assistance from clinicians, and acute exacerbations and mortality^9–12^. Increasing the referral of poorly controlled asthma patients may increase GP review. GP asthma reviews can help differentiate between poorly managed asthma and cases of severe or difficult-to-treat asthma which will guide management options for patients^13^.

Although pharmacists may appropriately refer patients to their GP, in the absence of a means to track continuity of care, little is known about what happens to the patient following these referrals, including uptake of the referral and the subsequent impact on patient outcomes and their care. There have been attempts to quantify the number of GP referrals initiated, as well as evaluate the appropriateness and potential benefits of pharmacist-initiated GP referrals^14–19^; however, less is known about uptake and health outcomes of referrals. Based on available literature, patient uptake of pharmacist-initiated GP referrals is estimated to be between 12 and 92%, varying greatly under different clinical scenarios and study populations^20^.

The current research investigated the uptake and outcomes of pharmacist-initiated GP referrals during a cluster randomized control trial to investigate the effectiveness of a pharmacist-initiated Pharmacy Asthma Service (PAS) where GP referrals were a feature of both the intervention and comparator arm protocol^21^. Amongst patients who received a referral, we investigated predictors of uptake of the referral, and compared asthma-related health outcomes between patients who actioned the pharmacist’s referral *(action takers)* and those who did not *(action avoiders)*.

## Methods

Pharmacies from regional and metropolitan areas in New South Wales (NSW), Western Australia (WA), and Tasmania participated in the PAS trial. Pharmacies were randomly assigned to either the PAS arm or usual-care arm. Randomization was stratified according to State and remoteness index using the Pharmacy Access/Remoteness Index of Australia (PhARIA)^22–24^ and randomly assigned in a 1:1 ratio to PAS and the usual-care arm within each stratum. These ensured pharmacies were representative of the distribution of the Australian population^21^. Pharmacists received specialized training in both asthma theory and inhaler device technique to ensure they had the necessary knowledge and technical skills to deliver the PAS^25^. The education was hosted on the Pharmaceutical Society of Australia’s (PSA) online education platform and was accredited for continuing professional development (CPD)^25^. This research was approved by the Human Research Ethics Committees of The University of Sydney, Curtin University, and The University of Tasmania^26^.

All pharmacists and patients provided written and electronic informed consent. Additionally, all patients consented to the collection of their Medicare Benefits Schedule (MBS) and Pharmaceutical Benefits Scheme (PBS) data. The MBS is an Australian Government initiative that subsidizes select health services for Australian citizens^27^. Similarly, the PBS is an Australian Government initiative that subsidizes prescription medicines for Australian citizens^28^. A record of all PBS subsidized medicines purchased by each patient spanning 12 months prior to their entry into the trial and for the 12 months they participated in the trial was collected. Services Australia (formerly the Australian Department of Human Services) is acknowledged for supplying both MBS and PBS information. The data presented in this study represent only patients who completed the 12-month trial.

### Patient recruitment

Patients with uncontrolled asthma, as determined by a score ≥1.5 in the Asthma Control Questionnaire (ACQ)^29,30^, aged ≥18 years, and who were able to communicate with the pharmacist in English, were a regular patient of the pharmacy (receiving medications from that pharmacy for the previous 12 months) and managing their own medication (as determined by the pharmacist) were included if they were willing to participate.

Patients were excluded from the study if they had a high dependence on medical care (more than five morbidities and specialist care), were unable to manage their own medication (as determined by the pharmacist), and/or had a confirmed diagnosis of chronic obstructive pulmonary disorder (as reported by the patient) or a terminal illness.

### Patient referral pathways

**Fig. 1:**
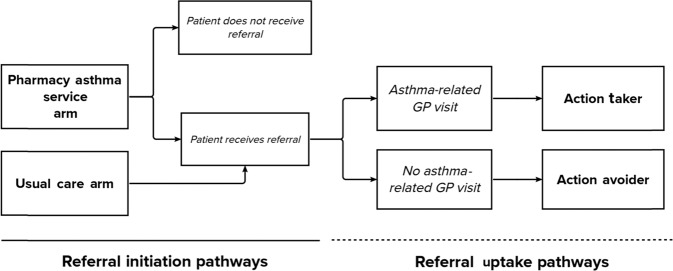
Referral initiation and uptake pathways taken within the Pharmacy Asthma Service trial. Initiation pathways (unbroken line); Referral uptake pathways (broken line).

Depending on the assigned arm of the patient’s pharmacy, patients proceeded into either the PAS or usual-care arm of the PAS trial (Table 1). Each arm incorporated different pharmacist-initiated GP referral pathways, as described below, and visualized in Fig. 1.

**Table 1:** Data collection time points and outcome measures.

		Baseline	Month 1	Month 6	Month 12
PAS arm		(In-pharmacy)	(In-pharmacy)	(Telephone)	(In-pharmacy)
	Asthma Control Questionnaire (ACQ)	x	x	x	x
	The Impact of Asthma on Quality-of-Life Questionnaire (IAQLQ)	x	x		x
	Rhinitis Control Assessment Test (RCAT)	x	x		x
	Demographic and asthma history data collected	x			
	Healthcare utilization (hospitalizations and emergency presentations)	x			x
	MBS and PBS data collected (12 months)	x			x
	Asthma action plan status				x
	Asthma management and medication changes		x	x	x
	PAS interventions delivered	x	x	x	x
	GP referral	If required	If required	If required	If required
Usual-care arm		(In-pharmacy)	(Telephone)		(Telephone)
	Asthma Control Questionnaire (ACQ)	x	x		x
	The Impact of Asthma on Quality-of-Life Questionnaire (IAQLQ)	x	x		x
	Rhinitis Control Assessment Test (RCAT)	x	x		x
	Demographic and asthma history data collected	x			
	Healthcare utilization (hospitalizations and emergency presentations)	x			x
	MBS and PBS data collected (12 months)	x			x
	Asthma action plan status				x
	Asthma management and medication changes		x		x
	GP referral	Compulsory	If required	If required	If required

After being screened for and being identified as having poorly controlled asthma, patients within the PAS arm attended three private face-to-face consultations with their pharmacist over a period of 12 months: at baseline, month 1, and month 12, with one additional telephone follow-up at month 6 to monitor progress and identify risks. The consultations provided education and counseling-based interventions targeting three key factors associated with uncontrolled asthma: (i) poor adherence^31,32^, characterized by underuse of preventer medication and/or overuse of reliever medication, (ii) suboptimal inhaler technique^22–24,33^, and/or (iii) uncontrolled allergic rhinitis^32–36^. Pharmacists were encouraged to provide GP referrals after each consultation if issues were identified with the patient’s asthma management.

After screening and being included if they had poorly controlled asthma, patients within the usual-care arm took part in one in-person initial consultation with their pharmacist where asthma and allergic rhinitis questionnaires were administered. Following this consultation, all patients were given a referral to their GP. Pharmacists then contacted patients by telephone at one month and 12 months after their initial consultation, to collect comparative data (no adherence, inhaler device technique, or allergic rhinitis interventions were prescribed within the usual-care arm protocol). Pharmacists were able to provide additional GP referrals to their patients at month 1 and month 12, based on patient needs.

### Referral initiation, uptake, and outcomes

The protocol required pharmacists to generate personalized referral letters for the patient’s GP using a template embedded into GuildCareNG™, a professional services software package operating in over 5000 pharmacies in Australia^37^. Records of each consultation and referral letter were created automatically in each patient’s GuildCareNG profile. Pharmacists in both arms of the study recorded whether a GP referral letter was issued to the patient upon completion of each session. All data were entered via trial-specific web-based data collection integrated with GuildCareNG.

To record uptake of referrals, pharmacists were prompted by the software at month 1, month 6 (PAS arm only), and month 12 to record if the patient had reported visiting their GP about their asthma since their last consultation.

If the patient had indicated at month 1, month 6 (PAS arm only) and/or month 12 that they had actioned the GP referral, the pharmacists asked the patient about any changes to their asthma management as a result. The pharmacist updated the patient’s GuildCareNG profile accordingly.

### Referral uptake

Based on patient’s uptake of the referral, all patients irrespective of trial arm allocation were categorized as either:

(i) *Action takers*—Patients who, upon the pharmacist’s advice, had visited their GP at least once for an asthma-related consultation during the 12-month trial period (Fig. 1).
(ii) *Action avoiders*—Patients who, despite their pharmacist’s advice, did not visit their GP for an asthma-related consultation during the 12-month trial period (Fig. 1).

To explore predictors of referral uptake, patient demographic and baseline variables were compared between *action takers* and *action avoiders*. These variables included the trial arm in which the patient participated, age, gender, work and education status, age at which the patient started experiencing asthma symptoms, and smoking status. Furthermore, data relating to the 12 months preceding the trial were also compared between the two patient groups and included whether the patient had a lung function test, an emergency presentation and/or hospital admission, and the total number of GP visits over the past 12 months as per MBS records. MBS data were used to calculate the total number of GP visits made by each participant during the 12 months preceding a patient’s entry into the trial and the 12 months during which the patient participated in the trial. For the purposes of this study, “total GP visits” were identified as all GP attendances, whether they were asthma-related or not. Baseline clinical measures were also compared: asthma control via the ACQ^29^, patient quality of life via the Impact of Asthma on Quality of Life Questionnaire (IAQLQ)^38^, allergic rhinitis control via the Rhinitis Control Assessment Test (RCAT)^39,40^, self-reported reliever use^21^, and adherence to preventer medication in the 12 months preceding the trial as per PBS data using the Proportion of Days Covered (PDC) method^41–43^. The PDC refers to the proportion of days in a given period of time covered by at least one asthma-preventer medication. The number of days covered is based on the dates a prescription was dispensed, the number of devices per script, the actuations per device and the participants prescribed dose in a given period of time^41–43^.

To determine if patient referral uptake was associated with differential asthma-related patient outcomes, a series of clinical outcome variables collected at month 12 were compared between *action takers* and *action avoiders* in each arm. These variables comprised asthma control as assessed via the ACQ^29^, patient quality of life via the IAQLQ^38^ and allergic rhinitis control via the RCAT^39,40^, patient self-reported reliever use^21^, and patient adherence to preventer medication during the 12-month trial period based on PBS data using the proportion of days covered (PDC) method^41–43^. It also included whether the patient had an asthma-related emergency presentation and/or hospital admission, whether a patient received a lung function test and the total number of GP visits the patient attended, based on MBS records during the trial period. In addition, whether patients were in possession of a current asthma action plan by the trial’s end.

### Data analysis

Cross-sectional data collected by the project-specific software was imported into SPSS^®^ Version 25, where descriptive statistics were applied. Data collection time points and outcome measures are illustrated in Table 1. Statistical associations were explored using either Pearson’s Chi-Square test or Fishers Exact Test when variable counts were below five for independent categorical variables. Mann–Whitney *U* test or Kruskal–Wallis tests were used for continuous variables. A *P* value of < 0.05 was considered statistically significant.

Following exploratory univariate analysis of variables associated with referral uptake, multivariate logistic regression was performed using variables with a univariate *P* value of < 0.20.

Six patients did not consent to the collection of MBS data, and thus mean estimates for total GP visits during the trial and in the 12 months preceding the trial are based on the available data. No data imputation was required, due to the forced entry functionality in GuildCareNG.

### Reporting summary

Further information on research design is available in the Nature Research Reporting Summary linked to this article.

## Results

### Referral initiation, uptake, and outcomes

In total, 58% of patients (*n* = 148) received at least one GP referral at baseline, month 1 or month 6 of the trial from their pharmacists. This included 100% (*n* = 111) of patients within the usual-care arm (the protocol required pharmacists to provide GP referrals to all patients) and 26% (*n* = 37) of patients within the PAS arm (the protocol allowed pharmacists to initiate GP referrals based on patient need) (Table 2).

**Table 2:** Patient referrals administered, self-reported uptake, and outcomes.

		PAS arm (*n* = 143), *n* (%)	Usual-care arm (*n* = 111), *n* (%)	*P* value	Total (*n* = *254*), *n* (%)
Patient received GP referral during trial		37 (25.9)^a^	111 (100.0)^a^	–	148 (58.3)
Patient visited the GP by month 12		31 (83.8)	84 (75.7)	0.305	115 (77.7)
Outcome Summary	Changes to therapy	24 (77.4)^	26 (31.0)	0.000*	50 (43.5)
	Medication ceased	7 (22.6)	8 (9.5)	0.041*	15 (30.0)
	New medication	13 (41.9)	16 (19.0)	0.006*	29 (58.0)
	Dose increase	6 (19.4)	9 (10.7)	0.157	15 (30.0)
	Dose decrease	4 (12.9)	2 (2.4)	0.034*	6 (12.0)
	Other	6 (19.4)	4 (4.8)	0.016*	10 (20.0)
Patient did not receive GP referral during trial		106 (74.1)	0 (0.0)	–	106 (74.1)
Patient visited the GP by month 12		72 (67.9)	–	–	72 (67.9)
Outcome summary	Changes to therapy	26 (36.1)^	–	–	26 (36.1)
	Medication ceased	8 (11.1)	–	–	8 (30.8)
	New medication	12 (16.7)	–	–	12 (46.2)
	Dose increase	8 (11.1)	–	–	8 (30.8)
	Dose decrease	5 (6.9)	–	–	5 (19.2)
	Other	5 (6.9)	–	–	5 (19.2)

*PAS* Pharmacy Asthma Service.

*Significant result.

^*P* **=** 0.000—comparing changes to therapy between those that received a referral versus those that did not.

*P* values were calculated using either a Pearson’s Chi-Square test or Fishers Exact Test when variable counts were below five for independent categorical variables. A significance level of *P* < 0.05 was used for all statistical procedures.

^a^In accordance with the trial protocol, PAS arm referrals were initiated based on patient need. All patients within the Usual-care arm were issued a GP referral at the completion of their baseline contact to ensure appropriate duty of care.

Among all PAS patients who reported visiting their GP during the trial for an asthma-related consultation, irrespective of a referral being issued, a referral was significantly more likely than the absence of a referral to generate medication changes (77%, *n* = 24 vs 36%, *n* = 26) (*X*^2^ = 19.624, df = 1, *P* < 0.0001). This could not be assessed within the usual-care arm as all patients received GP referrals. Further, a significantly higher proportion of PAS patients who received a referral and visited their GP during the trial, reported changes to their asthma management (77%, *n* = 24) when compared to the usual-care arm, patients who visited their GP following a referral (31%, *n* = 26) (*X*^2^ = 21.304, df = 1, *P* < 0.0001).

Among all patients who received a GP referral (*n* = 148), 115 (78%) were considered *action takers* (visited their GP regarding their asthma), and 33 (22%) were *action avoiders*.

Forty-four percent (*n* = 50) of *action takers* reported that the GP initiated a change to their asthma therapy. Reported changes were the addition of a new medicine (58%, *n* = 29), cessation of medicine (30%, *n* = 15), dose increase (30%, *n* = 15), dose decrease (12%, *n* = 6), or other change (20%, *n* = 10).

### Exploring predictors of patient referral uptake

Univariate analysis (Table 3) demonstrated that a higher proportion of patients residing in accessible locations were *action takers* compared to patients residing in highly accessible or rural or remote localities (OR = 8.775, 95% CI: 2.236, 34.444). A higher proportion of patients who developed asthma before the age of 35 years or after the age of 55 years were *action takers* (OR = 0.104, 95% CI: 0.012, 0.930). Additionally, in the 12 months preceding the trial, a larger proportion of *action takers* had self-reported a hospital admission (OR = 0.140, 95% CI: 0.018, 1.082), or an emergency presentation (OR = 0.343, 95% CI: 0.112, 1.051), and according to MBS data, had a higher total GP visit count (OR = 1.081, 95% CI: 1.011, 1.156) in comparison with *action avoiders*.

**Table 3:** Logistic regression model exploring the association between patient characteristics and baseline clinical markers to referral uptake.

		*Action taker*^b^ (*n* = *115*)	*Action avoider*^c^ (*n* = 33)	Odds ratio (univariable) OR (CI, *P*)	Adjusted odds ratio (multivariable) OR (CI, *P*)
Patient remoteness^a^	Highly accessible	66 (76.7)	20 (23.3)	1.97 (1.060–8.321, 0.038)	2.15 (0.683–6.758, 0.191)
	Accessible	39 (90.7)	4 (9.3)	8.78 (2.236–34.444, 0.002)	7.61 (1.744–33.324, 0.007)
	Moderately accessible, remote, very remote	10 (52.6)	9 (47.4)	–	–
Age of asthma onset	0–5 years of age	32 (82.1)	7 (17.9)	0.25 (0.029–2.232, 0.216)	0.302 (0.030–3.019, 0.308)
	6–15 years of age	18 (69.2)	8 (30.8)	0.13 (0.014–1.105, 0.216)	0.150 (0.015–1.509, 0.107)
	16–34 years of age	32 (78.0)	9 (22.0)	0.20 (0.023–1.698, 0.138)	0.221 (0.024–2.048, 0.184)
	35–55 years of age	15 (65.2)	8 (34.8)	0.10 (0.012–0.930, 0.043)	0.128 (0.013–1.222, 0.074)
	Above 55 years of age	18 (94.7)	1 (5.3)	–	–
Total GP visits as per MBS data *[Median (Q1-Q3)]*		8.6 (5.5–16.0)	6.0 (4.0–12.0)	1.08 (1.011–1.156, 0.22)	1.09 (1.005–1.171, 0.037)
Hospital admission for asthma in 12 months prior to trial	No	94 (74.6)	32 (25.4)	0.14 (0.018–1.082, 0.060)	0.25 (0.025–2.483, 0.237)
	Yes	21 (95.5)	1 (4.5)	–	–
Emergency presentation for asthma in 12 months prior to trial	No	82 (73.9)	29 (26.1)	0.34 (0.112–1.051, 0.061)	0.88 (0.223–3.458, 0.853)
	Yes	33 (89.2)	4 (10.8)	–	–

^a^Remoteness is based on PhARIA classifications. Participating pharmacies were identified as either “highly accessible” (PhARIA Category 1), “accessible” (PhARIA Categories 2 and 3) or “moderately accessible, remote or very remote” (PhARIA Categories 4, 5, and 6)^64–67^.

^b^Action takers: Patients who, upon the pharmacist’s advice, had visited their GP at least once for an asthma-related consultation during the 12-month trial period.

^c^Action avoiders: Patients who, despite their pharmacist’s advice, did not visit their GP for an asthma-related consultation during the 12-month trial period.

Multivariate logistic regression identified a patient residing in accessible localities (OR = 7.614, 95% CI: 1.744, 33.234) and a greater total GP visit count (OR = 1.085, 95% CI: 1.005, 1.171) as variables significantly associated with *action-taking* behavior.

### Referral uptake and asthma-related health outcomes

A higher proportion of *action takers* (54%, *n* = 62) had an up-to-date asthma action plan at month 12 compared to *action avoiders* (21%, *n* = 7) (*X*^2^ = 11.018, df = 1, *P* = 0.001). Although *action avoiders* stated they had not consulted their GP regarding their asthma during the trial period, based on MBS data, 88% (*n* = 29) of *action avoiders* visited their GP for other reasons during the trial. *Action takers* had a significantly higher total of unspecified GP visits (mean = 13 visits), according to MBS data, during the 12-month trial compared to *action avoiders* (mean = 8 visits) (*P* = 0.034). All other outcomes variables (asthma control, patient quality of life, allergic rhinitis control, patient self-reported reliever use, patient adherence to preventer medication, asthma-related emergency presentations, and/or hospital admissions and whether a patient received a lung function test during the trial period) were comparable among *action takers* and *action avoiders*.

## Discussion

This investigation explored the uptake and outcomes of pharmacist-initiated GP referrals for patients with poorly controlled asthma in a pharmacist-delivered asthma management service program. Over half of the cohort received at least one pharmacist-initiated GP referral during the trial period, and subsequently over three-quarters of those referred saw their GP about their asthma (*action takers)*. Amongst *action takers*, there was a greater likelihood of GP review, as changes to therapy/asthma management were reported for over 40% of *action takers*, and a significantly higher number of *action takers* were in possession of a current asthma action plan at the trial end compared to those who did not action the pharmacist’s referral (*action avoiders*). Patient referral uptake, however, did not translate to better therapeutic outcomes amongst patients during the 12-month trial period, as other patient-related asthma outcomes—asthma control, quality of life, rate of hospitalization, and allergic rhinitis control—were not statistically different at the end of the trial between *action takers* and *action avoiders*. This lack of a statistical difference may be due to the sample size as the study was not powered to assess these differences.

Regression analysis determined that patient remoteness was a strong predictor of *action-taking* behavior following a referral. A higher proportion of patients from accessible areas were *action takers* compared to patients residing in highly accessible or moderately accessible, remote, very remote localities. The stratifications utilized in this exploration were based on the degree of remoteness, both geographic and professional, of pharmacies^44^. Accessible areas are defined by ‘some restrictions to accessibility to some goods, services and opportunities for social interaction’ and include inner regional areas of Australia as opposed to highly accessible areas where there is relatively unrestricted access to goods, services, and social opportunities such as in major cities^44^. Moderately accessible, remote, and very remote areas refer to outer regional, remote, and very remote areas of Australia and are defined by significantly restricted, very restricted or very little access to good, services and social opportunities^44^. It could be that a combination of access and stronger relationships between health professionals and patients in inner regional centers could encourage a patient to engage with their GP^45–47^. In moderately accessible, remote, and very remote localities, however, there is lower access to healthcare, which may prevent patients from seeking care^48^. It is within these areas that allied health professionals, including pharmacists, can play an important role in helping to safeguarding health within these communities^49^. Further research is needed to confirm this finding and explore the potential behaviors or relationship issues that prevent patients within highly accessible areas from seeking further care from general practitioners.

In addition, regression analysis found that a higher number of GP visits (as per MBS records) in the 12 months preceding the trial was another predictor of *action-taking* behavior following a referral. *Action takers* had a significantly higher number of total GP visits (as per MBS records) than *action avoiders* and thus are already routinely seeking care regularly which makes it an obvious driver of future action-taking tendencies.

The univariate analysis found a significantly higher proportion of *action takers* had incurred a hospital admission and/or emergency presentation in the 12 months preceding entry into the trial, when compared to *action avoiders*. Such occurrences may help to focus a person with asthma on the need to regain control of their disease and seek appropriate care, or could suggest more severe or poorly controlled illness. The analysis also determined that a higher proportion of patients who developed asthma before the age of 35 years and after the age of 55 years were *action takers*. This finding requires further exploration.

As would be expected, among patients who did consult the doctor, there was greater evidence of GP review, as a significantly higher proportion of *action takers* had an up-to-date asthma action plan at month 12 than *action avoiders*. It is estimated that only ~28% of the Australian community with asthma are in possession of an action plan^50^, which is considerably lower than the 54% possession achieved amongst *action takers*. Additionally, almost half of *action takers* reported changes to asthma therapy following a GP visit.

It appears that patient uptake of pharmacist-issued GP referrals was somewhat higher in the PAS arm patients compared to the usual-care arm (84% versus 76%, respectively). However, these differences were not statistically significant. Interestingly, in total, a similar proportion of PAS arm patients (whether they received a referral) visited their GP for an asthma-related consultation compared to usual-care arm patients over the duration of the trial (72% versus 76%). This similarity may be due to the small sample size, or that being in the trial indirectly prompted patients to see their GP regarding their asthma management. Supporting literature is limited and often study specific regarding uptake of pharmacist-initiated GP referrals. In a 2014 review, regarding pharmacist-administered diabetes and cardiovascular referrals, patient uptake varied from 12 to 92% depending on the study^20^. Differences in method of measurement of referral uptake, intervention intensity preceding a pharmacist-initiated GP referral, and condition being evaluated make it difficult to compare findings to the existing literature. Interestingly, the studies reporting higher uptake within the 2014 review, including Edwards^51^, and Peterson et al.^52^ reported 92.3% and 82.7% uptake, respectively, based on cardiovascular risk assessments, and these values were similar to the current study. Both of the literature studies were screening and education-based interventions and uptake was measured by patient follow-up^51,52^. In comparison, a similar cardiovascular screening and education intervention conducted by Olenak et al in 2003 only measured a 41% referral uptake upon follow-up of over 70% of patients^53^. In comparison, an intervention purely based on screening and referral for diabetes, conducted by Krass et al. in 2006 led to referral uptake of up to 56.4% of patients^54^. Thus, in future, to determine the optimal processes for referral uptake, similar methodology/measurements should be used. A significantly higher proportion of referrals administered to PAS arm patients resulted in therapy changes upon visiting a GP when compared to the usual-care arm patients. Within the PAS arm, when given the option to refer, after a suite of interventions, only one-quarter of patients were referred to the GP for higher levels of care outside the scope of a pharmacist. However, in terms of referral outcome, these referrals led to medication changes in a significantly higher proportion of PAS arm patients compared to the usual-care arm patients who were all given referrals. This may indicate that a referral following a formal review of patient factors affecting asthma control and a more pointed referral to the GP identifying issues requiring review has more impact than the protocol-driven referral given to patients in the usual-care arm. Thus, pharmacist input may provide additional value into identifying patients’ management issues and/or suggestions for improvements combined with GP care.

Whereas identification of asthma issues and formal review by a pharmacist resulted in high uptake and changes in medication, innovative methods are needed to engage those patients who did not follow up referral. Our data demonstrate that around one in five patients did not seek medical review for their asthma, despite being identified by their pharmacist as having poorly controlled asthma. Additionally, almost all of the those not seeking medical review for their asthma saw their GP for other reasons during the 12-month trial. We need to investigate any perception deficits, financial and personal factors that delay patients seeking care for their asthma and/or recognizing the importance of optimal management^55^. Even then, we still cannot be assured of understanding the human psyche and the unique cognitive and emotional factors that govern how patients make decisions about their health and prioritize health issues. Such an exploration relies on deeper explorations through psychological, philosophical, and behavioral teachings^56–59^.

Pharmacists are expected to triage patients to other health professionals/medical care when their assessments of a patients’ health or medication use status mandates medical oversight. Behavioral aspects influence this process; a pharmacist needs to “provide” the referral, a patient needs to “action” it by taking it to their referred medical health professional, most often a GP, and the GP then needs to “act upon” the referral by making any necessary changes to the patient’s treatment. Patients within the PAS trial possibly had a higher ‘perceived susceptibility’ about their vulnerability to the impact of asthma^60,61^, and were indeed more likely to demonstrate action-taking behavior. GPs appeared more likely to initiate therapy changes when receiving patients from the PAS intervention arm possibly as they perceived the assessment being more in-depth. Pharmacists referred patients as this was a stipulated part of the PAS protocol, it may be that some of them may not do so within usual-care practices. In addition, the aim of this study was to investigate the impact of pharmacists’ services on patient asthma outcomes. Thus, it may be suggested that future projects need to be designed to specially diagnose behavioral stimuli that propel referral related action-taking in all three parties concerned (patient, pharmacist, and medical providers) and the interplay between meso- and macro-environmental factors that can facilitate or impede the process. In such explorations. frameworks such as the social ecological model or the COM-B (capability, opportunity, or motivation factors that impact behaviors) would well serve as underpinning frameworks^62,63^.

Outcome measures regarding the uptake and outcomes of pharmacists-administered GP referrals and subsequent asthma-related consultations, patient hospitalizations, and emergency presentations relied on patient self-report, and thus are subject to recall bias. Pharmacists were also required to record the administering of a formal patient referral to the GP. It is not certain whether informal prompts were given during consultations that may have encouraged patients to see their GP in the absence of a formal recorded referral.

GP review is essential for optimal asthma management, and our findings illustrate that 78% of patients with poorly controlled asthma took up their pharmacist’s referral for medical review. There was evidence of medication changes among those who acted upon their referral. Thus, there is an opportunity for pharmacists and GPs to collaborate to optimize patient care.

## Supplementary information

Reporting Summary

## Data Availability

The project-generated datasets used and/or analyzed during this study are available from the corresponding author upon request. Please note access to raw Pharmaceutical Benefits Scheme and Medicare Benefits Schedule data is subject to approval by Services Australia prior. Requests should be directed to sarah.serhal@sydney.edu.au.
