# Supplementary material for: Patient uptake and outcomes following pharmacist-initiated referrals to general practitioners for asthma review
Source: NPJ Prim Care Respir Med. 2022 Nov 17;32:53. doi: 10.1038/s41533-022-00315-6 (PMC9668973; doi:10.1038/s41533-022-00315-6)
Supplement: Supplementary file 1 — Reporting Summary [file 41533_2022_315_MOESM1_ESM.pdf]

nature portfolio

Corresponding author(s): Sarah Serhal

Last updated by author(s): 8.08.2022

Reporting Summary

Nature Portfolio wishes to improve the reproducibility of the work that we publish. This form provides structure for consistency and transparency in reporting. For further information on Nature Portfolio policies, see our Editorial Policies and the Editorial Policy Checklist.

Statistics

For all statistical analyses, confirm that the following items are present in the figure legend, table legend, main text, or Methods section.

n/a	Confirmed	
☐	☒	The exact sample size (*n*) for each experimental group/condition, given as a discrete number and unit of measurement
☐	☒	A statement on whether measurements were taken from distinct samples or whether the same sample was measured repeatedly
☐	☒	The statistical test(s) used AND whether they are one- or two-sided<br>*Only common tests should be described solely by name; describe more complex techniques in the Methods section.*
☐	☒	A description of all covariates tested
☐	☒	A description of any assumptions or corrections, such as tests of normality and adjustment for multiple comparisons
☐	☒	A full description of the statistical parameters including central tendency (e.g. means) or other basic estimates (e.g. regression coefficient) AND variation (e.g. standard deviation) or associated estimates of uncertainty (e.g. confidence intervals)
☐	☒	For null hypothesis testing, the test statistic (e.g. *F*, *t*, *r*) with confidence intervals, effect sizes, degrees of freedom and *P* value noted<br>*Give P values as exact values whenever suitable.*
☐	☒	For Bayesian analysis, information on the choice of priors and Markov chain Monte Carlo settings
☐	☒	For hierarchical and complex designs, identification of the appropriate level for tests and full reporting of outcomes
☐	☒	Estimates of effect sizes (e.g. Cohen's *d*, Pearson's *r*), indicating how they were calculated

Our web collection on statistics for biologists contains articles on many of the points above.

Software and code

Policy information about availability of computer code

Data collection	*Provide a description of all commercial, open source and custom code used to collect the data in this study, specifying the version used OR state that no software was used.*
Data analysis	*Provide a description of all commercial, open source and custom code used to analyse the data in this study, specifying the version used OR state that no software was used.*

For manuscripts utilizing custom algorithms or software that are central to the research but not yet described in published literature, software must be made available to editors and reviewers. We strongly encourage code deposition in a community repository (e.g. GitHub). See the Nature Portfolio guidelines for submitting code & software for further information.

Data

Policy information about availability of data

All manuscripts must include a data availability statement. This statement should provide the following information, where applicable:

- Accession codes, unique identifiers, or web links for publicly available datasets
- A description of any restrictions on data availability
- For clinical datasets or third party data, please ensure that the statement adheres to our policy

The project generated datasets used and/or analyzed during the current study are available from the corresponding author on reasonable request. Please note

access to raw Pharmaceutical Benefits Scheme and Medicare Benefits Schedule data is subject to approval by Services Australia prior. Requests should be directed to sarah.serhal@sydney.edu.au.

Human research participants

Policy information about studies involving human research participants and Sex and Gender in Research.

Reporting on sex and gender	Gender was self-reported within this text. Individual level data has not been included.
Population characteristics	Participants had uncontrolled asthma, as determined by a score ≥1.5 in the Asthma Control Questionnaire (ACQ), were aged ≥18 years, and were able to communicate with the pharmacist in English, they were a regular patient of the pharmacy (receiving medications from that pharmacy for the previous 12 months) and managing their own medication (as determined by the pharmacist) were included if they were willing to participate.
Recruitment	Patients were recruited by pharmacy staff from their regular pharmacy if they fulfilled the following criteria. Patients with uncontrolled asthma, as determined by a score ≥1.5 in the Asthma Control Questionnaire (ACQ),26, 27 aged ≥18 years, and who were able to communicate with the pharmacist in English, were a regular patient of the pharmacy (receiving medications from that pharmacy for the previous 12 months) and managing their own medication (as determined by the pharmacist) were included if they were willing to participate. Patients were excluded from the study if they had a high dependence on medical care (more than five morbidities and specialist care), were unable to manage their own medication (as determined by the pharmacist), and/or had a confirmed diagnosis of chronic obstructive pulmonary disorder (as reported by the patient) or a terminal illness.
Ethics oversight	This research was approved by the Human Research Ethics Committees of The University of Sydney, Curtin University and The University of Tasmania and registered with the Australian New Zealand Clinical Trials Registry (ACTRN12618000313235).

Note that full information on the approval of the study protocol must also be provided in the manuscript.

Field-specific reporting

Please select the one below that is the best fit for your research. If you are not sure, read the appropriate sections before making your selection.

☐ Life sciences ☒ Behavioural & social sciences ☐ Ecological, evolutionary & environmental sciences

For a reference copy of the document with all sections, see nature.com/documents/nr-reporting-summary-flat.pdf

Behavioural & social sciences study design

All studies must disclose on these points even when the disclosure is negative.

Study description	Study presents quantitative analysis.
Research sample	Participants had uncontrolled asthma, as determined by a score ≥1.5 in the Asthma Control Questionnaire (ACQ), were aged ≥18 years, and were able to communicate with the pharmacist in English, they were a regular patient of the pharmacy (receiving medications from that pharmacy for the previous 12 months) and managing their own medication (as determined by the pharmacist) were included if they were willing to participate. This study used trial data collected from a larger RCT.
Sampling strategy	Participants were recruited by community pharmacists.
Data collection	Data was collected using a study specific data collection software.
Timing	July 2018 to February 2020
Data exclusions	No data was excluded.
Non-participation	254 participants had a full 12 months of data collection out of 381 participants that completed baseline. 127 participants were loss to follow up.
Randomization	Pharmacies were randomized into intervention or control arm. Depending on the assigned arm of the patient's pharmacy, patients proceeded into either the PAS or usual-care arm of the trial.

Reporting for specific materials, systems and methods

We require information from authors about some types of materials, experimental systems and methods used in many studies. Here, indicate whether each material, system or method listed is relevant to your study. If you are not sure if a list item applies to your research, read the appropriate section before selecting a response.

Materials & experimental systems

n/a	Involved in the study
☒	☐ Antibodies
☒	☐ Eukaryotic cell lines
☒	☐ Palaeontology and archaeology
☒	☐ Animals and other organisms
☐	☒ Clinical data
☒	☐ Dual use research of concern

Methods

n/a	Involved in the study
☒	☐ ChIP-seq
☒	☐ Flow cytometry
☒	☐ MRI-based neuroimaging

Clinical data

Policy information about clinical studies

All manuscripts should comply with the ICMJE guidelines for publication of clinical research and a completed CONSORT checklist must be included with all submissions.

Clinical trial registration

ACTRN12618000313235

Study protocol

https://www.anzctr.org.au/Trial/Registration/TrialReview.aspx?id=374558&isReview=true] or in a previously published manuscript: Serhal S, Saini B, Bosnic-Anticevich S, Krass I, Emmerton L, Bereznicki B, et al. A Targeted Approach to Improve Asthma Control Using Community Pharmacists. Frontiers in Pharmacology. 2021;12:798263 DOI: 10.3389/fphar.2021.798263.

Data collection

Data collection occurred within community pharmacists. Data was collected from July 2018 to February 2020.

Outcomes

The current research investigated the uptake and outcomes of pharmacist-initiated GP referrals during a cluster randomized-controlled trial to investigate the effectiveness of a pharmacist-initiated Pharmacy Asthma Service (PAS) where GP referrals were a feature of both the intervention and comparator arm protocol.

Amongst patients who received a referral, we investigated predictors of uptake of the referral, and compared asthma-related health outcomes between patients who actioned the pharmacist's referral (action takers) and those who did not (action avoiders).

Based on patient's uptake of the referral, all patients irrespective of trial arm allocation were categorized as either:
(i) Action takers - Patients who, upon the pharmacist's advice, had visited their GP at least once for an asthma-related consultation during the 12-month trial period.
(ii) Action avoiders – Patients who, despite their pharmacist's advice, did not visit their GP for an asthma-related consultation during the 12-month trial period.

Exploring predictors of referral uptake
To explore predictors of referral uptake, patient demographic and baseline variables were compared between action takers and action avoiders. These variables included the trial arm in which the patient participated, age, gender, work and education status, age at which the patient started experiencing asthma symptoms, and smoking status. Furthermore, data relating to the 12 months preceding the trial were also compared between the two patient groups and included whether the patient had a lung function test, an emergency presentation and/or hospital admission, and the total number of GP visits over the past 12 months as per MBS records. MBS data were used to calculate the total number of GP visits made by each participant during the 12 months preceding a patient's entry into the trial and the 12 months during which the patient participated in the trial. For the purposes of this study, 'total GP visits' were identified as all GP attendances, whether they were asthma related or not. Baseline clinical measures were also compared: asthma control via the ACQ26, patient quality of life via the Impact of Asthma on Quality of Life Questionnaire (IAQLQ)38, allergic rhinitis control via the Rhinitis Control Assessment Test (RCAT)39, 40, self-reported reliever use, 21 and adherence to preventer medication in the 12 months preceding the trial as per PBS data using the Proportion of Days Covered (PDC) method.41-43

Comparison of asthma-related health outcomes
To determine if patient referral uptake was associated with differential asthma-related patient outcomes, a series of clinical outcome variables collected at month 12 were compared between action takers and action avoiders in each arm. These variables comprised asthma control as assessed via the ACQ,26 patient quality of life via the IAQLQ38 and allergic rhinitis control via the RCAT,39, 40 patient self-reported reliever use21 and patient adherence to preventer medication during the 12-month trial period based on PBS data using the proportion of days covered (PDC) method.41-43 It also included whether the patient had an asthma-related emergency presentation and/or hospital admission, whether a patient received a lung function test and the total number of GP visits the patient attended, based on MBS records during the trial period. Additionally, whether patients were in possession of a current asthma action plan by the trials end.
